# Teaching a neural network to attach and detach electrons from molecules

**DOI:** 10.1038/s41467-021-24904-0

**Published:** 2021-08-11

**Authors:** Roman Zubatyuk, Justin S. Smith, Benjamin T. Nebgen, Sergei Tretiak, Olexandr Isayev

**Affiliations:** 1 Department of Chemistry, Carnegie Mellon University, Pittsburgh, PA USA; 2 Theoretical Division, Los Alamos National Laboratory, Los Alamos, NM USA; 3 Center for Integrated Nanotechnologies, Los Alamos National Laboratory, Los Alamos, NM USA

**Keywords:** Cheminformatics, Computational chemistry, Quantum chemistry

## Abstract

Interatomic potentials derived with Machine Learning algorithms such as Deep-Neural Networks (DNNs), achieve the accuracy of high-fidelity quantum mechanical (QM) methods in areas traditionally dominated by empirical force fields and allow performing massive simulations. Most DNN potentials were parametrized for neutral molecules or closed-shell ions due to architectural limitations. In this work, we propose an improved machine learning framework for simulating open-shell anions and cations. We introduce the AIMNet-NSE (Neural Spin Equilibration) architecture, which can predict molecular energies for an arbitrary combination of molecular charge and spin multiplicity with errors of about 2–3 kcal/mol and spin-charges with error errors ~0.01e for small and medium-sized organic molecules, compared to the reference QM simulations. The AIMNet-NSE model allows to fully bypass QM calculations and derive the ionization potential, electron affinity, and conceptual Density Functional Theory quantities like electronegativity, hardness, and condensed Fukui functions. We show that these descriptors, along with learned atomic representations, could be used to model chemical reactivity through an example of regioselectivity in electrophilic aromatic substitution reactions.

## Abstract (web summary)

Quantum mechanical calculations of molecular ionized states are computationally quite expensive. This work reports a successful extension of a previous deep-neural networks approach towards transferable neural-network models for predicting multiple properties of open shell anions and cations.

## Introduction

A large body of research in the field of chemistry is concerned with the flow and behavior of electrons, which gives rise to important phenomena such as making and breaking chemical bonds. Quantum chemistry (QC) provides a mathematical framework for describing the behavior of atomistic systems through the solution of the Schrödinger equation, allowing for a detailed description of charge distribution and molecular energetics. QC provides the tools to accurately construct the potential energy surface (PES) of molecules, i.e., energy as a function of molecular geometry. Density Functional Theory (DFT) framework often underpins the methods of choice for such calculations when working with medium-size molecules by providing a good balance between accuracy and computational cost. Unfortunately, standard DFT methods for the treatment of the N-electron system typically require ~*O*(N^3^) numerical cost. This cubic scaling has become a critical challenge that limits the applicability of DFT to a few hundred atom systems. This also limits the accessibility of longer dynamical simulation time scales, which are critical for simulating certain experimental observables. Consequently, a lot of progress has been made in the development of interatomic potentials providing a complex sought out PES functional (geometry -> energy) using machine learning (ML)^1,2^, which have been applied to a variety of systems^3–8^.

Deep-neural networks (DNN)^9,10^ are a particular class of ML algorithms proven to be universal function approximators^11^. These DNNs are perfectly suitable to learn a representation of the PES for molecules. There are multiple distinct DNN models for ML potentials reported in the literature. They could be divided into two groups. The original Behler-Parrinello (BP)^12^ and its modifications ANI^13,14^ and TensorMol^15^ rely on 2-body (radial) and 3-body (angular) symmetry functions to construct a unique descriptor of atomic environment for a particular atom, then use a DNN to predict atomic properties as a function of that descriptor. Other models, for example, HIP-NN^16^, DTNN^4^, SchNet^17^, and PhysNet^18^ use non-invariant radial symmetry functions or interatomic distances and iteratively construct a representation of the atomic environment through message-passing techniques^19^.

The ANAKIN-ME (ANI) method^13,20^ is one example of a technique for building transferable DNN-based molecular potentials. The key components of ANI models are the diverse training data set^21^ and BP type descriptors^12^ with modified symmetry functions^13^. The ANI-1ccx data set was built from energies and forces for ~60K small organic molecules containing 5 and 0.5 million non-equilibrium molecular conformations calculated at DFT and high-fidelity Coupled Clusters (CCSD(T)) levels, respectively^21^. Test cases showed the ANI-1ccx model to be chemically accurate compared to the reference Coupled Cluster calculations and exceeding the accuracy of DFT in multiple applications^14^. Finally, the AIMNet (Atoms-In-Molecules neural Network) architecture, a chemically inspired, modular deep-neural network molecular potential improves the performance of ANI models for long-range interactions and continuum solvent effects^8^.

The physical properties of molecular systems are often labeled as intensive or extensive properties. This nomenclature relates to the dependency of the property upon the size of the system in question^22^. The notation has been introduced by Tolman over one hundred years ago^23^. Some studies have used ML for intensive properties^24–29^ independent of the system size, which poses challenges to ML techniques due to spatial non-locality and long-range interactions.

In this work, we examine how DNN models like ANI and AIMNet can be applied to predicting intensive properties like electron attachment (electron affinity) and electron detachment (ionization potential). The conventional wisdom would be to fit different ML potentials for every quantum-mechanical state (neutral, cation, and anion). QM calculations for ionized states of the molecule are typically more expensive due to the unrestricted Hamiltonian formalism and subsequent spin polarization of orbitals. Therefore, we seek to answer a critical question: Can we fuse information from different molecular charge states to make ML models more accurate, general, and data-efficient? With the success of deep learning in many applications involving complex multimodal data, this question can be addressed by learning different states of the molecules with one common ML model, and the goal is to use the data in a complementary manner toward learning a single complex problem. We explore two synergistic strategies for joint modeling: multitask learning^24,30^ and data fusion. One of the main advantages of joint learning is that a hierarchical representation can be automatically learned for each state, instead of individually training independent models. In addition to electron attachment and detachment energies, we also choose to learn spin-polarized charges for every state reflecting quantum mechanics of the wavefunctions. This choice of properties is deliberate, as it allowed us to compute reactivity descriptors such as philicity indices and Fukui functions based on conceptual Density Functional Theory (c-DFT) theory^31,32^. c-DFT, or Chemical Reactivity Theory, is a powerful tool for the prediction, analysis, and interpretation of chemical reactions^33,34^. Here all c-DFT indexes were computed directly from the neural network without additional training that permitted us to bypass quantum mechanical calculations entirely.

## Results

**Fig. 1:**
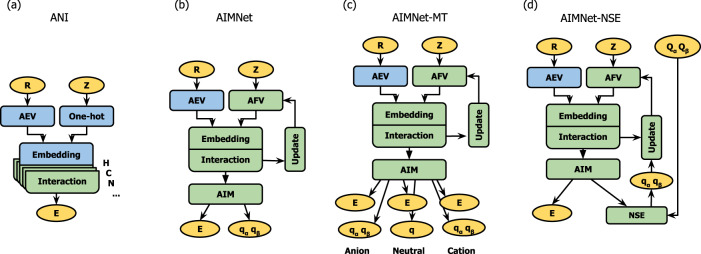
Neural network architectures explored in this work. Models from literature: **a** ANI^13^, **b** AIMNet^8^. Here each model is separately trained for neutral species, cations, and ions. Models introduced in this work: **c** AIMNet-MT: a multitask model jointly trained on all data which concurrently predicts energies and charges for neutral species as well as cations and ions; and **d** AIMNet-NSE, a Neural Charge Equilibration model which is capable to re-distribute spin-polarized atomic charges according to a given molecular spin charges and predicts energy for the specified (arbitrary) spin state of the molecule. The yellow blocks show input data (coordinates **R**, atomic numbers **Z**, and total molecular spin charge **Q**) and output quantities (energies **E** and spin-polarized charges **q**). The green blocks denote trainable modules, and the blue blocks are fixed encodings.

High-dimensional neural networks (HDNNs)^12^ rely on the chemical bonding nearsightedness (‘chemistry is local’) principle by decomposition of the total energy of a chemical system into atomic contributions. For each atom in the molecule, HDNN models encode the local environment (a set of atoms within a pre-defined cutoff radius) as a fixed-size vector and use it as an input to a feed-forward DNN function to infer individual atomic contribution to the total energy. The ANI model (Fig. 1a) transforms coordinates **R** of the atoms in the molecule into atomic environment vectors (**AEV**s): a set of translation, rotation, and permutation invariant two-body radial ${g}_{{ij}}^{(r)}$ (gaussian expansion of interatomic distances) and three-body angular ${g}_{{ijk}}^{(a)}$ (joint gaussian expansion of average distances to a pair of neighbors and cosine expansion of angle to those atoms) symmetry functions, where index *i* corresponds to a “central” atom and *j* and *k* refer to the atoms from its environment. Using the information of atomic species types **Z**, the **AEV**’s are reduced in a permutation-invariant manner into the **Embedding** vectors ***G****,* which encode both geometrical and type information of the atomic environment. The ANI model uses the concatenation of the sums of ${g}_{{ij}}^{(r)}$ and ${g}_{{ijk}}^{(a)}$, which correspond to a distinct chemical type of neighbor, or a combination of the types for two neighbors. This is equivalent to multiplication of the matrices ${{{{{{\boldsymbol{g}}}}}}}_{{{{{{\boldsymbol{i}}}}}}}^{({{{{{\boldsymbol{r}}}}}})}$ and ${{{{{{\boldsymbol{g}}}}}}}_{{{{{{\boldsymbol{i}}}}}}}^{({{{{{\boldsymbol{a}}}}}})}$ with rows composed of **AEV**’s, and corresponding matrices ***A***^***(r)***^ and ***A***^***(a)***^ composed with one-hot (categorical) encoded atom or atom-pair types:

1 $${{{{{{\boldsymbol{G}}}}}}}_{{{{{{\boldsymbol{i}}}}}}}=\left\{{{{{{{\boldsymbol{g}}}}}}}_{{{{{{\boldsymbol{ i}}}}}}}^{\left({{{{{\boldsymbol{r}}}}}}\right){{\top }}}{{{{{{\boldsymbol{A}}}}}}}^{\left({{{{{\boldsymbol{r}}}}}}\right)},{{{{{{\boldsymbol{g}}}}}}}^{\left({{{{{\boldsymbol{a}}}}}}\right){{\top }}}{{{{{{\boldsymbol{ A}}}}}}}^{\left({{{{{\boldsymbol{a}}}}}}\right)}\right\}$$

This definition of the HDNN models suffers from the “curse of dimensionality” problem. Namely, the size of ***G*** depends on the number of unique combinations of atomic species included in parametrization (size of vectors in ***A***^***(a)***^). Also, since the information about the type of the “central” atom is not included in ***G***, it uses multiple independent DNNs defined for each atom type (${{{{{{\mathcal{F}}}}}}}^{({{{{{\mathcal{Z}}}}}}_{{{i}}})}$) to model **Interactions** of the atom with its environment and outputs atomic energy ${E}_{i}$:

2 $${E}_{i}={{{{{{\mathcal{F}}}}}}}^{({{{{{{\mathcal{Z}}}}}}}_{{{i}}})}\left({{{{{\boldsymbol{G}}}}}}_{{{{{\boldsymbol{i}}}}}}\right)$$

The AIMNet model (Fig. 1b) was developed to address the dimensionality issue with the ANI model. Instead of one-hot encoding of atomic species, it uses learnable atomic feature vectors (**AVF**s) ***A*** in Eq. 1. The **AFV** vectors encode similarities between chemical elements. This approach eliminates the dependence of the size of **Embedding** layer on the number of parametrized chemical species. The AIMNet model utilizes the idea of multimodal learning, making a simultaneous prediction of different atomic properties from several output heads attached to the common layer of multi-layer neural nets. This layer is enforced to capture the relationships across multiple learned modalities and serves as a joint latent representation of atoms in the molecule. Therefore, we call this layer an **AIM** vector. Finally, the architecture of AIMNet has a specific implementation of message passing through updating the **AFV** based on neighbor atoms atomic environments. This way, the model operates iteratively, at each iteration *t* predicting atomic properties ***P*** and updated features ***A***, using the same (shared across iterations) neural network function ${{{{{\mathcal{F}}}}}}$:

3 $$\{{P}_{i}^{t},{{{{{{\boldsymbol{A}}}}}}}_{i}^{t+1}\}{{{{{\mathscr{=}}}}}}{{{{{\mathcal{F}}}}}}\left({{{{{{\boldsymbol{G}}}}}}}_{{{{{{\boldsymbol{i}}}}}}}^{t}{{{{{\boldsymbol{,}}}}}}\,{{{{{{\boldsymbol{A}}}}}}}_{{{{{{\boldsymbol{i}}}}}}}^{t}\right)$$

The approach has an analogy with a solution of one-electron Schrodinger equation with self-consistent field (SCF) iterations, where one-electron orbitals (**AFV** in case of AIMNet) adapt to the potential introduced by other orbitals in the molecule (embedding vectors ***G*** in case of AIMNet). Though there is no convergence guarantee for AIMNet due to the absence of the variational principle, in practice statistical errors decrease and converge at *t* = 3 being an empirical observation.

The AIMNet and ANI models do not use total molecular charge and therefore could not discriminate between different charge states of the same conformer. The straightforward way to obtain reasonable predictions is to train separate models for neutral, anionic, and cationic species. Since the AIMNet model works well in multitask regime^8^, we also design an AIMNet architecture that simultaneously predicts energies and spin-polarized atomic charges with multiple output heads from the same **AIM** layer for a pre-defined set of charge states (AIMNet-MT, Fig. 1c). All three states share the same **AFV** representation, **Interaction**, and **Update** blocks. This setting allows us to evaluate if the common feature representations can capture correlations across different states and, if possible, take advantage of that.

In this paper, we introduce an extension to the AIMNet architecture which allows the model to predict energy, properties, and partial atomic charges for a specified state based on total molecular charge and spin multiplicity (or, alternatively, total *α* and *β* spin charges) given as input for the model. The key component of the new model is the Neural Spin-charge Equilibration unit (NSE, Fig. 1d), which makes prediction of partial spin-polarized atomic charges ${\widetilde{q}}^{s}$ and atomic weight factors ${f}^{s}$ (conceptually related to atomic Fukui functions, ∂q/∂Q) from the **AIM** layer using fully-connected NN output head. The factors ${f}^{s}$ are used to re-distribute atomic spin charges such as their sum is equal to the specified total molecular spin charges:

4 $${q}_{i}^{s}={\widetilde{q}}_{i}^{s}+\frac{{f}_{i}^{s}}{\mathop{\sum }\limits_{j=1}^{N}{f}_{j}^{s}}\left({Q}^{s}-\mathop{\sum }\limits_{j=1}^{N}{\widetilde{q}}_{j}^{s}\right)$$

where index *s* corresponds to spin-component of the charge density, $\widetilde{q}$ and $q$ are initial and re-normalized charges, *N* is number of atoms and $Q$ total is the total charge of the molecule. The consequent **Update** block injects normalized atomic charges into the **AFV** vector. This way, during the next AIMNet iteration, the information about charge distribution will be used in the **Embedding** block. We should note, that for the AIMNet and AIMNet-MT models the sum of atomic charges is not necessarily an integer, but rather is very close to the total integer molecular charge due to errors in atomic charge predictions. However, for the AIMNet-NSE model, the charges are conserved and add up to the total molecular charge by construction.

A summary of the performance for all four models is presented in Table 1. Vertical ionization potentials (IP) and electron affinities (EA) were computed directly from the corresponding differences of energies of neutral and charged states:

5 $${{{{{\rm{IP}}}}}}={{{E}}}_{{{{{{\rm{cation}}}}}}}-{{{E}}}_{{{{{{\rm{neutral}}}}}}};\,{{{{{\rm{EA}}}}}}={{{E}}}_{{{{{{\rm{neutral}}}}}}}-{{{E}}}_{{{{{{\rm{anion}}}}}}}$$

**Table 1:** Root-mean-square errors (RMSEs) in kcal/mol for total molecular energies and vertical ionization potentials (IP) and electron affinities (EA).

Model	Test data set	Cation	Neutral	Anion	IP	EA
ANI	Ions-12	8.4	5.1	5.0	9.4	6.9
	Ions-16	10.8	4.4	4.9	11.0	5.9
	Ions-16 (ens5)	10.0	4.0	4.6	10.2	5.3
AIMNet	Ions-12	4.1	3.7	3.0	4.7	4.4
	Ions-16	6.3	3.2	3.4	6.5	4.0
	Ions-16 (ens5)	5.3	**2.6**	**2.8**	5.3	**3.1**
	ChEMBL-20 (ens5)	12.8	5.3	6.0	9.2	2.9
AIMNet-MT	Ions-12	3.5	3.4	2.8	4.1	3.9
	Ions-16	5.4	3.0	3.2	5.5	3.5
	Ions-16 (ens5)	4.9	**2.5**	**2.7**	5.0	**3.0**
	ChEMBL-20 (ens5)	13.0	4.3	5.4	10.3	3.0
AIMNet-NSE	Ions-12	3.6	3.4	2.9	4.1	3.9
	Ions-16	3.9	3.1	3.1	4.1	3.6
	Ions-16 (ens5)	**3.4**	**2.5**	**2.6**	**3.5**	**3.0**
	ChEMBL-20 (ens5)	**4.0**	**3.4**	**3.8**	**2.7**	**2.4**

Best results are marked in bold.

Results obtained for the individual models and an ensemble of 5 models (ens5) on validation subset of Ions-12, and on Ions-16 and ChEMBL-20 external test sets.

The prediction errors are evaluated on the Ions-12 (up to 12 non-H atoms) data set which provides a measure of the performance of the model with respect to the data points similar to those used for training. On the other hand, errors on Ions-16 (13–16 non-H atoms) can be seen as a more appropriate testbed that is probing generalization capabilities of the model across the unknown chemical and conformational degrees of freedom (i.e., unseen molecules). Further, we evaluate the performance of the models on the data set of equilibrium conformations of neutral drug-like molecules ChEMBL−20 (13–20 non-H atoms) as a realistic example application of the model. We report root-mean-square errors (RMSE), rather than more popular in the field^5,17,35^ mean absolute errors (MAE). MAE is less sensitive to severe prediction errors and could often mislead about the generalization capabilities of the models.

While ANI models are known to achieve state-of-the-art performance^14,36^ on conformational energies and reaction thermochemistry in drug-like molecules, the problem addressed here is challenging due to the presence of charged species. Similar to our previous results for neutral molecules^8^, all AIMNet flavors substantially improve upon ANI, especially for the total energy of cations and vertical IPs. The original ANI model does not include explicit long-range interactions. All interactions are described implicitly by the neural network; therefore, the interactions described by the model do not extend beyond the AEV cutoff distance (*R*_cut_ = 5.2 Å in this work). Since the ANI model performs well on neutral molecules and is completely short-sighted and has no capability to perform charge equilibration either explicitly or implicitly, we use it as a baseline for comparison. Because both extra electrons (in case of anions) and holes (in case of cations) are spatially delocalized, the non-local electrostatics extends beyond the cutoff distance and spatially spans over the molecule.

**Fig. 2:**
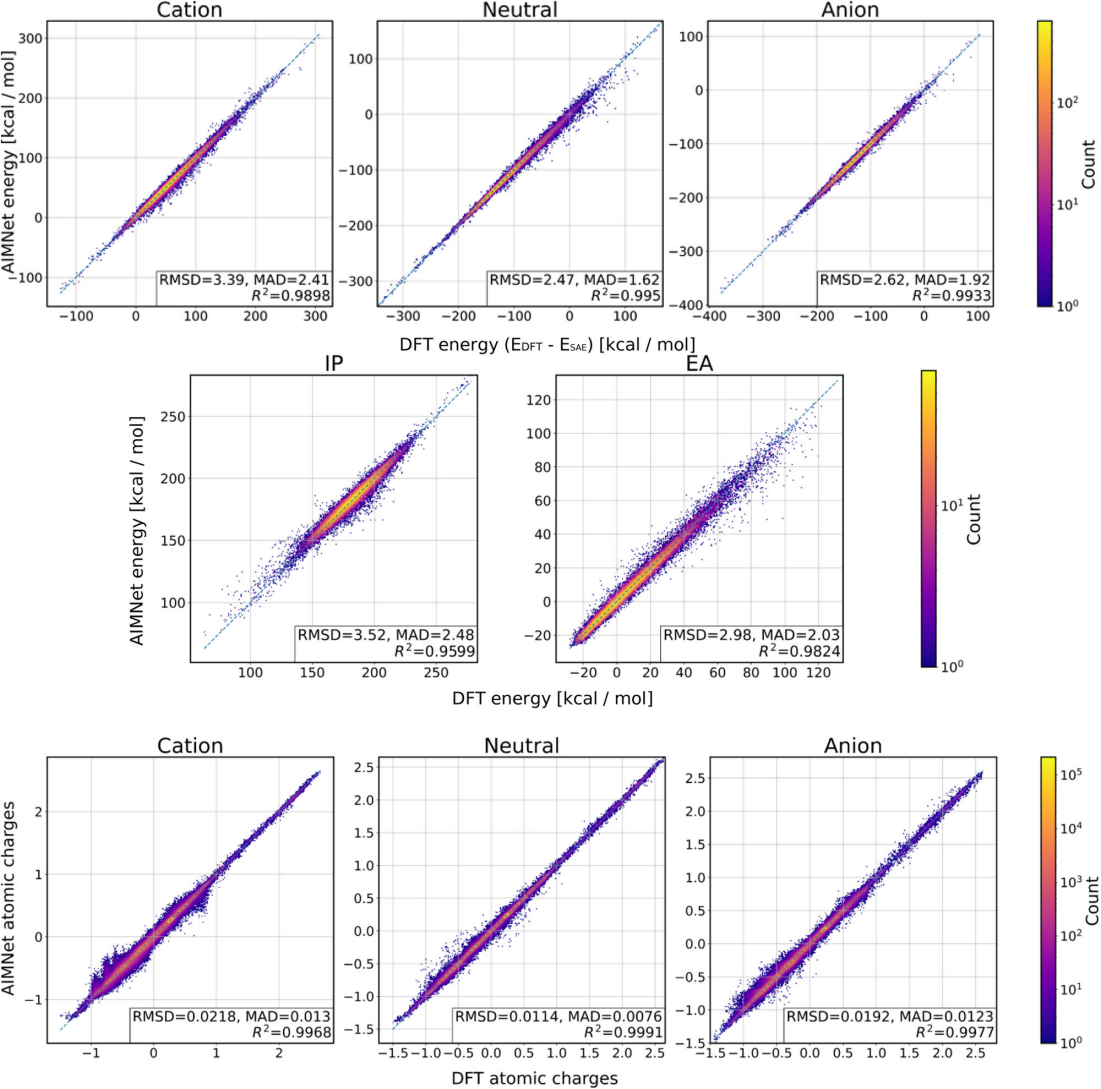
Performance evaluation for AIMNet-NSE model. Correlation between DFT PBE0/ma-def2-SVP and AIMNet-NSE predictions for total molecular energies (top row), non-equilibrium vertical ionization potentials (IP), and electron affinities (EA) (middle row) and NBO atomic charges (bottom row) calculated for three charge states for Ions-16 data set. DFT total energies were shifted by the sum of atomic self-energies (*E*_SAE_) to allow a comparison for molecules with different compositions. Element-specific *E*_SAE_ calculated using linear regression, correspond to average atomic energies in the entire training data set that include all charge states.

While the AIMNet and AIMNet-MT models show reasonable accuracy for neutral and anionic species, the errors for cations are few times larger, especially for the ChEMBL data set. This indicates the shortcoming in the extensibility of implicit charge equilibration with “SCF-like” passes. Overall, the data-fused AIMNet-MT model performs marginally better then separate AIMNet models for each charge state. Contrary, the AIMNet-NSE model with explicit charge equilibration shows consistent performance across charge states and molecule sizes, both for near and off-equilibrium conformers. The RMS errors on IP and EA values are approach 0.1 eV for optimized structures and to 0.15 eV for off-equilibrium geometries. Fig. 2 provides overall correlation plots for energies and charges as predicted by AIMNet-NSE model for Ions-16 data set. Please see Supplementary Figs. 3–5 for plots for similar plots produces with the other models. Note, since regression plots are colored by the density of points on the log scale, the vast majority of points are on the diagonal line. The AIMNet-NSE models consistently provide the same level of performance across the energy range of 400 kcal/mol (~17 eV) without noticeable outliers. The model is able to learn atomic charges up to 0.01*e* (electron, elementary charge) for neutral molecules and 0.02*e* for ions as shown in Fig. 2 (also see Supplementary Table 2). Table 1 also compares the performance of individual models to the performance of their ensemble prediction (marked as “ens5”). In principle, model ensembling is always desirable and, on average, provide a performance boost of 0.5 kcal/mol for all energy-based quantities.

The AIMNet-NSE model has a superb utility for high-throughput applications. In this sense, it is interesting to compare this model with the excellent semi-empirical IPEA-xTB method^37^. The IPEA-xTB is a re-parametrization of GFN-XTB Hamiltonian to predict EA and IP values of organic and inorganic molecules. The re-parametrization aimed to reproduce PW6B95/def2-TZVPD results. The IPEA-xTB method was successfully used to make accurate predictions of electron ionization mass spectra^37^ and for high-throughput screening of polymers^38,39^. For medium-sized organic molecules, the AIMNet-NSE model brings the accuracy/computational performance ratio to the a new level. For the ChEMBL-20 data set, the RMSE of IPEA-xTB EA and IP vs PBE0/ma-def2-SVP are 4.6 and 10.6 kcal/mol, compared to AIMNet-NSE errors of 2.7 and 2.4 kcal/mol, respectively. Therefore, the AIMNet-NSE is considerably more accurate and at least two orders of magnitude faster than IPEA-xTB when running on similar hardware.

**Fig. 3:**
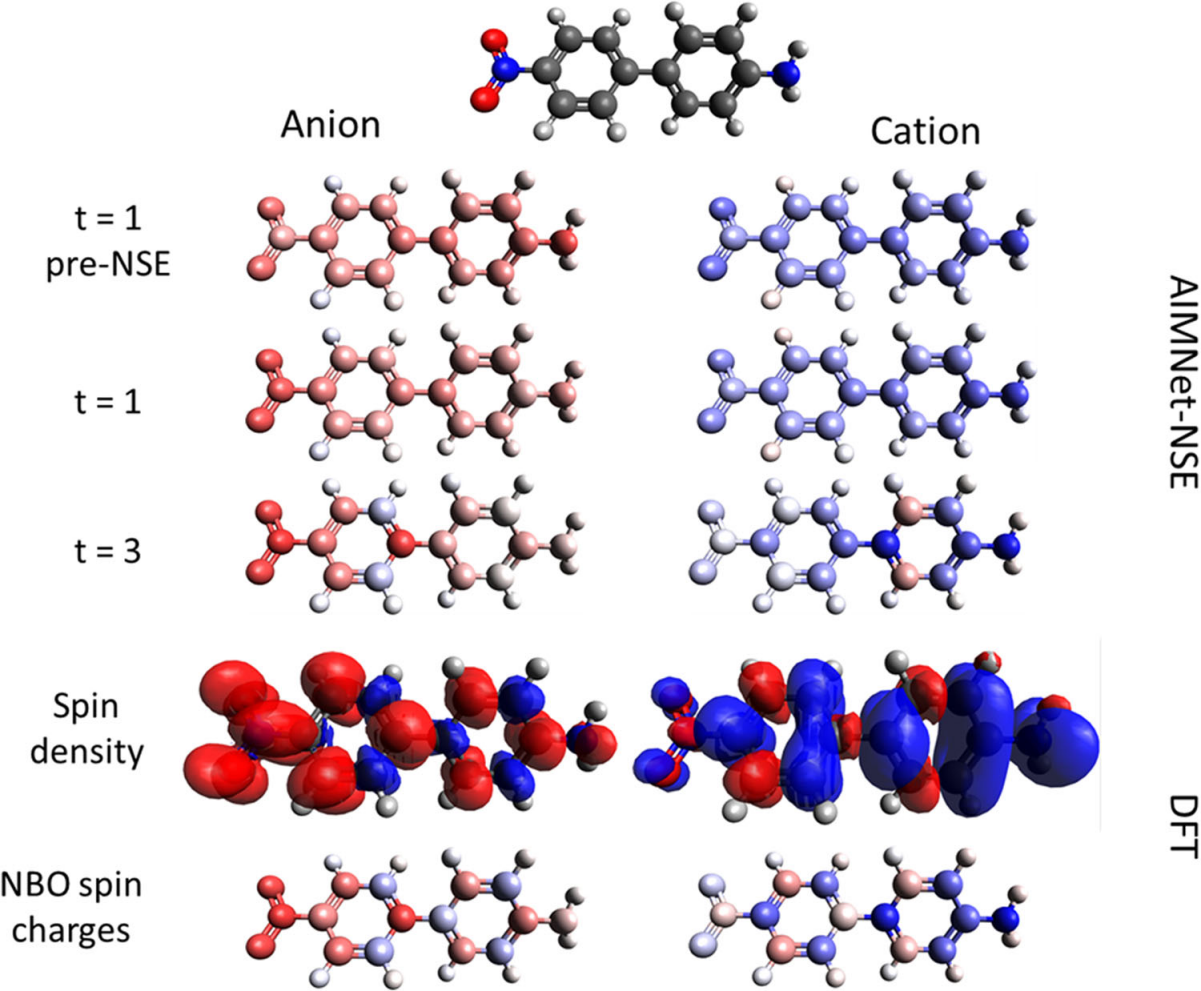
Neural Spin Equilibration (NSE) for ion-radicals of 4-amino-4′-nitrobiphenyl molecule. For the anion, colors correspond to spin electron atomic charges or density (*α* − *β*), while for cation to spin hole density (*β* − *α*), with red color corresponding to negative spin-charge. The parameter *t* corresponds to AIMNet iterative passes. For comparison, DFT (PBE0/ma-def2-SVP) spin-density and charges are depicted at the bottom.

To elucidate the importance of iterative “SCF-like” updates, the AIMNet model was evaluated with a different number of passes *t*. AIMNet with *t* = 1 is very similar to the ANI model. The receptive field of the model is roughly equal to the size of the AEV descriptor in ANI; and no updates were made to the AFV vector and atomic embeddings. Fig. 3 shows that the aggregated performance of prediction for energies improves with an increasing number of passes *t*. This trend is especially profound for cations. As expected, the accuracy of AIMNet with *t* = 1 is very similar or better compared to the ANI network. The second iteration (*t* = 2) provides the largest improvement in performance for all three states. After *t* = 3, the results are virtually converged. Therefore, we used *t* = 3 to train all models in this work. These observations for charged molecules are remarkably consistent with results for neutral species^8^.

Let us consider 4-amino-4′-nitrobiphenyl molecule as an illustrative example (Fig. 3). This is a prototypical optoelectronic system, where a π-conjugated system separates the electron-donating (NH_2_) and accepting (NO_2_) groups. These polar moieties underpin an increase in the transition dipole moment upon electronic excitation leading to two-photon absorption. The effect of donor-acceptor substitution is apparent from the ground-state calculations of the charge species where electron and hole in cation and anion, respectively, are shifted towards the substituent groups with strong delocalization across π orbitals of the aromatic rings. Fig. 3 illustrated the charge equilibration procedure in AIMNet-NSE models and compares it to DFT results. During the first pass, before charge normalization, the predicted densities are the same for anion and cation (note inverse color codes for anion and cation on Fig. 3), but after weighted normalization, the spin-charge density is already slightly shifted towards the nitro group in the anion and the amino group in the cation. At the same time spin charges on the hydrogen atoms does not change, as expected. After three iterations the AIMNet-NSE model correctly reproduces spin-density wave-like behavior with opposite phases for the cation and anion as predicted by DFT. There is no sign alternation for spin charge for 4, 4′ positions, however, the absolute value of spin-charge difference for these atoms is high. Overall, the AIMNet-NSE model predicts spin charges for non-hydrogen atoms of this molecule with MAE 0.03*e* for anion and 0.02*e* for cation. Notably, the 4-amino-4′-nitrobiphenyl molecule was neither part of the training nor validation data, exemplifying the new architecture’s ability to transfer spin-density predictions to completely unseen molecules.

In AIMNet-NSE, the physical meaning of the weights *f* (see Eq. 4) is related to atomic Fukui functions, $\partial {q}_{i}/\partial Q$, e.g., how much would atomic charge ${q}_{i}$ change with the change of total charge *Q*. In practice, the model would assign higher values of *f* to the atoms which tend to have different charges in different charge states of the molecule, for example, to aromatic and hereto atoms. The value of *f* also reflects the uncertainty in charge distribution predicted by the neural network. A somewhat related approach for weighted charge re-normalization was used previously^40^. It was based on charge prediction uncertainty estimated with ensemble of random forests, however without noticeable improvement in charge prediction accuracy. Our neural spin-charge equilibration method provides a simple and affordable alternative to other ML charge equilibration approaches^41–43^ based on QEq method which finds charge distribution by minimization of molecular Coulomb energy. While the QEq solution impose physics-based constraints for the obtained charge distribution, it is limited by the approximate form of Coulomb integral and could be computationally demanding due to the required matrix inversion operation.

The described neural charge equilibration could be an attractive alternative to popular charge equilibration schemes like EEM^44^, QEq^45^, and QTPIE^46^ that use simple physical relationships. They often suffer from transferability issues and might produce unphysical results. To our knowledge, this is a primary example where the ML model provides a consistent and qualitatively correct physical behavior between molecular geometry, energy, integral molecular charge, and partial atomic charges. Upon submitting this manuscript we learned about work by Xie^47^, where ML model built to predict energy as a function of electron populations in prototypical LiH clusters. Other schemes like BP^12^, TensorMol^15^, HIP-NN^48,49^, and PhysNet^18^ typically employ auxiliary neural network that predicts atomic charges from a local geometrical descriptor. Electrostatic interactions are computed with Coulomb’s law based on those charges. In principle, many effects can be captured by a geometrical descriptor, but it does not depend on the total charge and spin multiplicity of the molecule. Following the basic principles of quantum mechanics to incorporate such information successfully, the model should adapt according to changes in the electronic structure, preferably in a self-consistent way. This is exemplified here through the case of the AIMNet-NSE model.

### Case study for chemical reactivity and reaction prediction

As a practical application of AIMNet-NSE model, we demonstrate a case study on chemical reactivity and prediction of reaction outcomes. The robust prediction of the products of chemical reactions is of central importance to the chemical sciences. In principle, chemical reactions can be described by the stepwise rearrangement of electrons in molecules, which is also known as a reaction mechanism^50^. Understanding this reaction mechanism is crucial because it provides an atomistic insight into how and why the specific products are formed.

DFT has shown to be a powerful interpretative and computational tool for mechanism elucidation^51–54^. In particular, conceptual DFT (c-DFT) popularized many intuitive chemical concepts like electronegativity (*χ*) and chemical hardness (*η*)^55^. In c-DFT, reactive indexes measure the energy (*E*) change of a system when it is a subject to a perturbation in its number of electrons (*N*). The foundations of c-DFT were laid by Parr et al.^56^ with the identification of the electronic chemical potential *µ* and hardness η as the Lagrangian multipliers in the Euler equation. In the finite-difference formulation, these quantities could be derived from EA and IP values as

6 $$\mu =-\chi =\left(\frac{\partial E}{\partial N}\right)\approx -\frac{1}{2}\left({{{{{\rm{IP}}}}}}+{{{{{\rm{EA}}}}}}\right)$$

7 $$\eta =\left(\frac{{\partial }^{2}E}{\partial {N}^{2}}\right)\approx -\frac{1}{2}\left({{{{{\rm{IP}}}}}}-{{{{{\rm{EA}}}}}}\right)$$

The Fukui function *f*(*r*) is defined as a derivative of the electron density on the total number of electrons in the system. These global and condensed-to-atom local indexes were successfully applied to a variety of problems in chemical reactivity^57,58^. Using finite-difference approximation and condensed to atoms representation, Fukui functions for electrophilic (${f}_{a}^{-}$), nucleophilic (${f}_{a}^{+}$), and radical (${f}_{a}^{0}$) reactions are defined as:

8 $${f}_{a}^{-}={q}_{C}-{q}_{N};{f}_{a}^{+}={q}_{N}-{q}_{A};{f}_{a}^{\pm }=\frac{1}{2}({q}_{C}+{q}_{A})$$

Another useful c-DFT reactivity descriptor is the electrophilicity index given by

9 $$\omega ={\mu }^{2}/2\eta$$

as well as it’s condensed to atoms variants for electrophilic (${\omega }_{a}^{-}$), nucleophilic (${\omega }_{a}^{+}$) and radical (${\omega }_{a}^{\pm }$) attacks:^59^

10 $${\omega }_{a}^{-}=\omega {f}_{a}^{-};{\omega}_{a}^{+}=\omega {f}_{a}^{+};{\omega }_{a}^{\pm}=\omega {f}_{a}^{\pm }$$

**Fig. 4:**
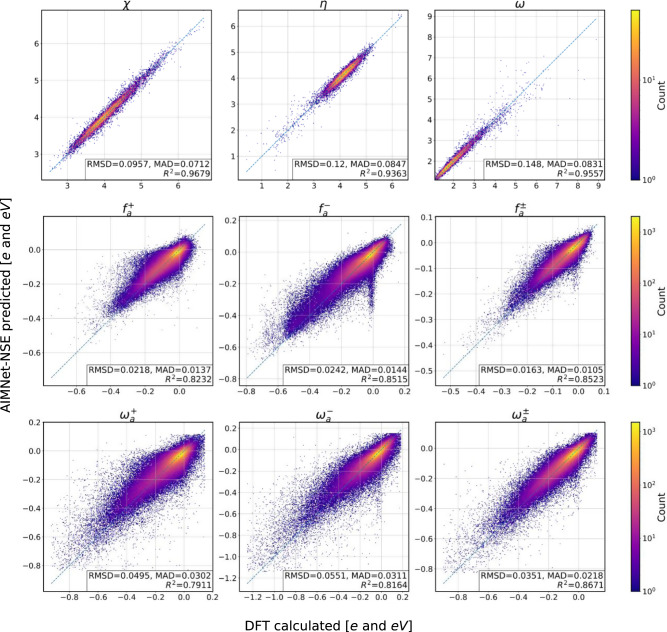
Conceptual DFT quantities predicted by the AIMNet-NSE model. Correlation between DFT PBE0/ma-def2-SVP and AIMNet-NSE predictions for electronegativity (*χ*), chemical hardness (*η*) and electrophilicity index ($\omega$), Fukui coefficients for nucleophilic (${f}_{a}^{+}$), for electrophilic (${f}_{a}^{-}$) and radical (${f}_{a}^{\pm }$) attacks and three corresponding condensed philicity indexes (*ω*_*a*_) for Ions-16 data set.

On the basis of the predicted with AIMNet-NSE vertical IPs, EAs, and charges, we could directly compute all listed c-DFT indexes. Fig. 4 displays the correlation plots for all nine quantities. The AIMNet-NSE model achieves an excellent quality of prediction of three global indexes with *R*^2^ ranging from 0.93 to 0.97. Condensed indexes are more challenging to predict, with philicity index (${\omega }_{a}^{+}$) being the hardest (*R*^2^ is 0.82). This is related to the overall larger errors in the cation energy predictions. Here we would like to emphasize again that none of these properties were part of the cost function or training data. The values were derived from the pre-trained neural network and therefore opens the possibility of direct modeling fully bypassing c-DFT calculations and wavefunction analysis. The accuracy of AIMNet-NSE predicted condensed indexes appears to be suitable to make a reliable prediction of reaction outcomes.

**Fig. 5:**
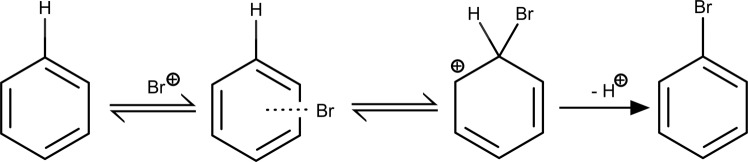
General mechanism of electrophilic aromatic substitution reaction. Bromination of the benzene molecule as a typical electrophilic aromatic substitution (EAS) reaction.

Let us exemplify the prediction of site selectivity for aromatic C–H bonds using electrophilic aromatic substitution (EAS) reaction. The EAS reaction is a standard organic transformation. Its mechanism involves the addition of an electrophile to the aromatic ring to form a σ-complex (Wheland intermediate) followed by deprotonation to yield the observed substitution product (Fig. 5). The reactivity and regioselectivity of EAS would generally depend on the ability of the substituents to stabilize or destabilize a σ-complex.

Recently EAS attracted significant attention from computational studies due to its importance in late-stage functionalization (LSF) for the drug development process^60^. A direct and numerically very expensive approach to EAS selectivity predictions is to calculate all transition states on the complete path from reactants to products. A popular approach called RegioSQM achieves high site prediction accuracy based on enumeration and calculation of σ-complex with semi-empirical quantum mechanical calculations^61^.

Table 2 lists the accuracy of regioselectivity prediction with recently published methods using data from ref. ^60^. A random forest (RF) model with DFT TPSSh/Def2-SVP derived descriptors like charges (*q*), bond orders (BO), Fukui indexes, and solvent accessible surface (SAS) achieves 90% accuracy on the validation data (note different DFT methodology used for this study and for training our DNNs). This model relies on QM calculations of reagents but does not require searching σ-complexes. When QM descriptors are combined with RegioSQM, the RF classifier exhibits an excellent performance of 93%. While the RegioSQM model is accurate, it is slow for high-throughput screening. A modest data set of a few hundred molecules takes about two days to complete on a multicore compute node. Very recently, Weisfeiler–Lehman Neural Network (WLNN) was suggested to predict site selectivity in aromatic C–H functionalization reactions^62^. This model was trained on 58,000 reactions from the Reaxys database and used RDKit molecular descriptors. WLNN achieves an accuracy approaching 90% for the prediction of EAS regioselectivity.

**Table 2:** Compilation of results for EAS regioselectivity prediction with different approaches.

Descriptors	ML Model	Validation accuracy	Test accuracy
q, BO, SAS, *f*_*−*_	RF^a^	0.899	
q, BO, SAS, *f*_*−*_, RegioSQM	RF^a^	0.931	0.876
Reaxis data, molecular descriptors	Weisfeiler–Lehman Neural Net^b^	0.895	0.836
$\omega ,{\omega }_{a}^{-}$, *AIM* vector	RF (present work)	0.906	0.850

^a^Results from ref. ^60^.

^b^Results from ref. ^62^.

We used AIMNet-NSE to calculate Fukui coefficients and atomic philicity indexes. We also added the AIM layer of the query atom in cation-radical form of the molecule as an additional set of descriptors. The size of the AIM layer is smaller (144 elements) than the training data set size (602 data points). The use of cross-validation scores and the random forest method generally mitigates any overfitting issues. As we argued before^8^ the multimodal knowledge residing inside the AIM layer could be exploited as an information-rich feature representation. The RF classifier trained with AIMNet-NSE descriptors displays an excellent performance of 90% on the validation set and 85% on the test set. While obtained predictions for the electrophilic aromatic substitution reaction are only marginally better than previously reported values, our model achieve six orders of magnitude computational speedup since no quantum mechanical simulations are necessary.

## Discussion

We recently witnessed that machine learning models trained to quantum-mechanical data achieve formidable success in quantitative predictions of ground-state energies and interatomic potentials for common, typically charge-neutral organic molecules. Nevertheless, a quantitative description of complex chemical processes involving reactions, bond breaking, charged species, and radicals remains an outstanding problem for data science. The conceptual challenge is a proper description of spatially delocalized electronic density (which strongly depends on molecular conformation) and accounting for long-range Coulombic interactions stemming from the inhomogeneously distributed charges. These phenomena appear as a consequence of the quantum-mechanical description of delocalized electronic wavefunctions. Consequently, representation of spatially non-local, frequently intensive molecular properties is problematic for common neural nets adapting local geometric descriptors. The recently developed AIMNet neural network architecture addresses this challenge via an iterative message passing-based process, which ultimately captures complex latent relationships across atoms in the molecule.

In the present work, we introduced the AIMNet-NSE architecture to learn a transferrable potential for organic molecules in arbitrary charge states. For neutral, cation-radical and anion-radical species, the AIMNet-NSE model achieves consistent 3–4 kcal/mol accuracy in predicting energies of larger molecules (13–20 non-H atoms), even though it was only trained small molecular up to 12 non-H atoms. In addition to energy, the AIMNet-NSE model achieve state-of-the-art performance in the prediction of intensive properties. It demonstrates accuracy of about 0.10–0.15 eV for vertical electron affinities and ionization potentials across a broad chemical and conformational space.

The key ingredients that allow the AIMNet-NSE model to achieve such a high level of accuracy are (i) multimodal learning, (ii) joint information-rich representation of atom in a molecule that is shared across multiple modalities, and (iii) Neural Spin-Charge Equilibration (NSE) block inside the neural network. In contrast to the standard geometric descriptors, we have highlighted the importance of incorporating adaptable electronic information into ML models. Essentially the AIMNet-NSE model serves as a charge equilibration scheme. AIMNet-NSE brings ML and physics-based models one step closer by offering a discrete, physically correct dependence of system energy with respect to a total molecular charge and spin states.

As a side benefit, it can provide a high-quality estimate of reactive indexes based on conceptual DFT and reliable prediction of reaction outcomes. Overall, demonstrated flexible incorporation of quantum mechanical information into the AIMNet structure and data fusion exemplify a step toward developing a universal single neural net architecture capable of quantitative prediction of multiple properties of interest. As we show in our case studies the AIMNet-NSE model appears as a fast and reliable method to compute multiple properties like ionization potential, electron affinity, spin-polarized charges, and a wide variety of conceptual DFT indexes. It potentially emerges as a drop-in replacement calculator in a myriad of potential applications where high computational accuracy and throughput are required.

## Methods

### Data set

For the training data set, we randomly selected about 200k neutral molecules from the UNICHEM database^63^ with molecule size up to 16 “heavy” (i.e., non-hydrogen) atoms and set of elements {H, C, N, O, F, Si, P, S, and Cl}. We choose molecular dynamics (MD) as a fast and simple method to explore molecular PESs around their minima. Thermal fluctuations of atoms in MD simulations allow for the near-equilibrium sampling of molecular conformational space. Similar approaches have been explored in previous reports^13,21^. Notably, all traditional molecular force fields are designed to describe closed-shell molecules only. Therefore, to overcome this limitation, we choose a quantum mechanically derived force field (QMDFF ^64^) as an efficient method to construct system-specific and charge-specific mechanistic potential for a molecule. We relied on the GFN2-xTB^65^ tight-binding model to obtain minimum conformation, force constants, charges, and bond orders that are needed for the QMDFF model.

**Fig. 6:**
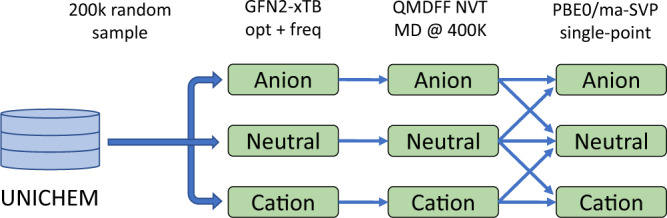
The workflow for data set generation for the neutral and charged molecular species. The molecules to construct the training data set were sampled from the UNICHEM database. Potential energy surface was sampled with GFN2-XTB and QMDFF molecular dynamics. Reference QM energies and charges obtained at PBE0/ma-def2-SVP level. The lines represent data flow during data generation.

The workflow to generate molecular conformations is summarized in Fig. 6. Starting from SMILES representations, we generated a single 3D conformation for each molecule using the RDKit ^66^ library. The molecule in each of three charge states (i.e., neutral, cation and anion) was optimized using the GFN2-xTB method, followed by a calculation of force constants, charges, and bonds orders to fit molecule-specific QMDFF parameters. This custom force field was used to perform a 500 ps NVT MD run, with snapshots collected every 50 ps for the subsequent DFT calculations. For each snapshot, we performed several single-point DFT calculations with a charge for the molecule set to the value at which the MD was performed, as well as its neighboring charge state, i.e., −1, 0 for anions, −1, 0, +1 for neutral, and 0, +1 for cations (Fig. 6). This results in up to 70 single-point DFT calculations per molecule. For DFT calculations we selected PBE0/ma-def2-SVP level of theory as a reasonable compromise between accuracy and computational expenses. PBE0 is a non-empirical hybrid DFT that is widely used to compute molecular properties. Exact exchange and diffuse functions in the basis set are needed in order to describe anionic species. All DFT calculations were performed using the ORCA 4.0 package^67^. Atomic spin-polarized charges were calculated the NBO-7 software package^68^ for PBE0/ma-def2-SVP wavefunction.

We split all data into two subsets: Ions-12 data set contains 6.44 M structures with up to 12 heavy atoms of which 45%, 25%, and 30% are neutral, cations, and anions, respectively. Ions-16 data set has 295k structures of 13–16 non-hydrogen atoms size with 48%, 24%, and 26% of neutral, anionic, and cationic species, respectively. Please see Supplementary Table 1 and Figs. 1–2 for more details. We used Ions-12 data set for training and validation, whereas Ions-16 was utilized for testing. Ions-16 data set has larger, more complex structures and thus probes the model transferability.

For further evaluation of model performance, transferability, and extensibility we compiled a data set that should be close to real-world application. We randomly selected 800 of organic molecules from ChEMBL database^69,70^ with 13–20 non-hydrogen atoms, 100 per molecular size. The neutral state of each molecule was optimized with B97-3c composite DFT method^71^, then a single-point energy calculation using the same B97-3c method was performed for anion and cation radicals. The resulting data set, referred as ChEMBL-20, covers equilibrium conformations of “drug-like” molecules.

### Training protocol

The ANI model and AIMNet variants were trained using minibatch gradient descent powered by the Adam optimizer^72^. For training performance considerations, all minibatches were composed of molecules with the same number of atoms, to avoid padding. Proper data feed shuffling was achieved with the multi-GPU Data-parallel approach: gradients on model weights were averaged after 8 random batches were evaluated in parallel. The effective combined batch size was 2048. The training was performed on 8 Nvidia V100 GPUs, with a computational cost of about 200 s for the AIMNet-MT model and 130 s for the AIMNet-NSE model per epoch of Ions-12 data set with 6.4 M data points. We employ a reduce-on-plateau learning rate schedule, which leads to training convergence within 400–500 epochs.

The training objective was minimization of weighted multi-target mean squared error (MSE) loss function with included errors in energy and charge predictions. The AIMNet architecture shares weights of Embedding, Interaction blocks, and fully-connected output heads for all “SCF-like” iterative passes. The models were trained with 3 passes. The outputs from each pass were included into weight function, except for during training the AIMNet-NSE model. Due to the architecture of the AIMNet-NSE model during the first pass, it makes predictions without the use of information about the total spin charge. Therefore, for this model only, outputs from the two last passes were included in the loss function. Although all final predictions of AIMNet models were obtained with *t* = 3, we found it beneficial to restrain a network to give reasonably accurate results on earlier iterative passes, as it provides regularization to the model. Additional details about the loss function are given in the SI.

The baseline ANI and AIMNet models were trained independently for each of the three charge states of the molecules. For AIMNet-MT and AIMNet-NSE, joint training for all charge states was performed, and errors for each charge state were included in the loss function. The training was done against 5-fold cross-validation data splits. These five independent models were used to build an ensemble for more accurate predictions, denoted as “ens5” later in the text. All AIMNet model variants, as well as the ANI model, were implemented with the PyTorch framework^73^. The AIMNet-NSE model, example inference scripts, and test datasets are available in a public code repository at https://github.com/isayevlab/aimnetnse.

## Supplementary information

Supplementary Information

## Data Availability

The test datasets used this study are publicly available at 10.5281/zenodo.5007980.

## Code availability

The trained AIMNet-NSE models in and the code to reproduce this study is available at 10.5281/zenodo.5008270 and in GitHub at https://github.com/isayevlab/aimnetnse.
