# Supplementary material for: Teaching a neural network to attach and detach electrons from molecules
Source: Nat Commun. 2021 Aug 11;12:4870. doi: 10.1038/s41467-021-24904-0 (PMC8357920; doi:10.1038/s41467-021-24904-0)
Supplement: Supplementary file 1 — Supplementary Information [file 41467_2021_24904_MOESM1_ESM.pdf]

Supplementary Materials for

Teaching neural network to attach and detach electrons from molecules

*Roman Zubatyuk, Justin S. Smith, Benjamin T. Nebgen, Sergei Tretiak, Olexandr Isayev**

*Corresponding author. Email: olexandr@olexandrisayev.com

This PDF file includes:

Supplementary Table 1: Number of molecules and conformers in training and test datasets.

Supplementary Figure 1: Distribution of molecule sizes in training and test datasets.

Supplementary Figure 2: Distribution of molecular energies for training and test datasets.

Supplementary Table2: Root mean square errors (RMSEs) for atomic partial charge (DFT/MBIS) prediction for individual models and ensemble of 5 models (ens5) on Ions-12 test set and Ions-16 external set.

Supplementary Note 1: Loss function for the AIMNet models training.

Supplementary Figure 3: Correlation plots for predicted versus DFT total molecular energies for Ions-16 dataset.

Supplementary Figure 4: Correlation plots for predicted versus DFT ionization potentials (IP) and electron attachment energies (EA) for Ions-16 dataset.

Supplementary Figure 5: Correlation plots for predicted versus DFT MBIS partial atomic charges for Ions-16 dataset.

Supplementary Table 1: Number of molecules and conformers in training and test datasets.

	Cations	Neutrals	Anions
Molecules			
Ions-12	154,381	167,103	155,981
Ions-16	13,607	16,138	13,860
Conformers			
Ions-12	1,896,354	3,273,755	2,150,019
Ions-16	75,654	148,543	85,741

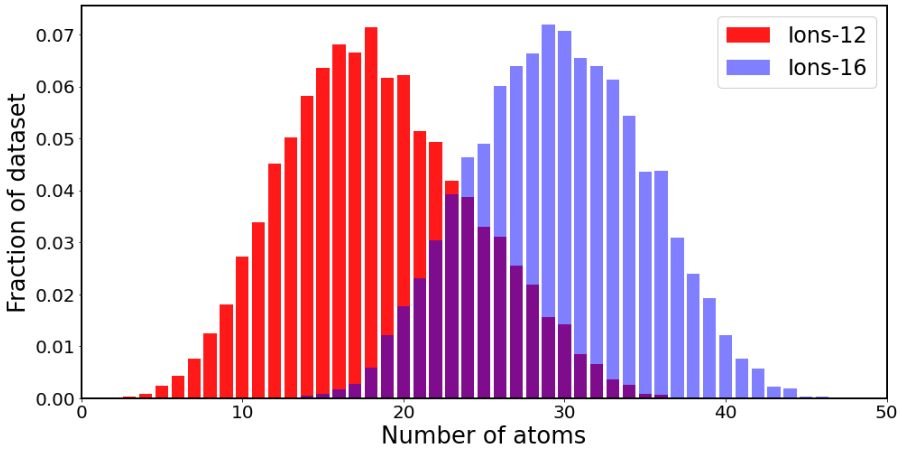

Supplementary Figure 1: Distribution of molecule sizes in training and test datasets.

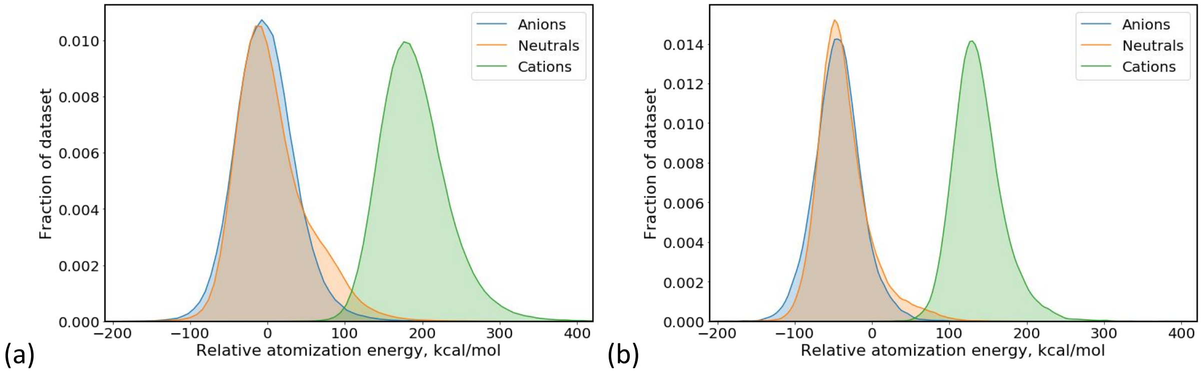

Supplementary Figure 2: Distribution of molecular energies for training and test datasets. Atomization energy defined as total DFT energy minus sum of atomic energies computed as average in the dataset.

Supplementary Table 2: Root mean square errors (RMSEs) for atomic partial charge (DFT/NBO) prediction for individual models and ensemble of 5 models (ens5) on Ions-12 test set and Ions-16 external set.

Model	Test Dataset	Partial charge RMSE		
		Cation	Neutral	Anion
AIMNet	Ions-12	0.028	0.017	0.026
	Ions-16	0.028	0.013	0.021
	Ions-16 (ens5)	0.026	0.011	0.019
	ChEMBL-20 (ens5)	0.031	0.016	0.042
AIMNet-MT	Ions-12	0.025	0.016	0.027
	Ions-16	0.024	0.013	0.022
	Ions-16 (ens5)	0.023	0.011	0.020
	ChEMBL-20 (ens5)	0.028	0.016	0.040
AIMNet-NSE	Ions-12	0.025	0.016	0.025
	Ions-16	0.024	0.013	0.021
	Ions-16 (ens5)	0.022	0.011	0.019
	ChEMBL-20 (ens5)	0.027	0.016	0.038

Supplementary Note 1: Loss function for the AIMNet models training.

The training objective was minimization of weighted multi-target mean squared error (MSE) loss function. For a molecule with N atoms it could be written as:

$$\mathcal{L} = \sum_{t=1}^{T} w_t(\mathcal{L}_E + w_q\mathcal{L}_q + w_{\tilde{q}}\mathcal{L}_{\tilde{q}}) \tag{S1}$$

$$\mathcal{L}_E = \frac{1}{N}\left(E - \hat{E}\right)^2 \tag{S2}$$

$$\mathcal{L}_q = \frac{1}{2N}\sum_{i=1}^{N}\sum_{s\subset\{\alpha,\beta\}}(q_i^s - \hat{q}_i^s)^2 \tag{S3}$$

$$\mathcal{L}_{\tilde{q}} = \frac{1}{2N}\sum_{i=1}^{N}\sum_{s\subset\{\alpha,\beta\}}(\hat{q}_i^s - \tilde{q}_i^s)^2 \tag{S3}$$

The loss functions include the weighted contributions from total energy prediction error $\mathcal{L}_E$ and spin-charges prediction error $\mathcal{L}_q$. For AIMNet-NSE we added squared difference between of predicted charges before and after normalization procedure (Eq. 4), to ensure that un-normalized charges are close to the normalized charges. For all AIMNet models, contributions from several "SCF-like" passes were included into the total loss with weights w_t. The weights w_q and $w_{\tilde{q}}$ were selected as 90 and 30 kcal mol^{-1} e^{-1} such as all energy and charges errors give approximately equal contributions to the loss function.

The models were trained with 3 passes, the outputs from each pass were included into weight function with weights w_t=[0.15, 0.25, 0.60] for AIMNet and AIMNet-MT and w_t=[0, 0.3, 0.7] for AIMNet-NSE.

Our experiments show that the final results are virtually the same regardless the choice of weights in the loss function.

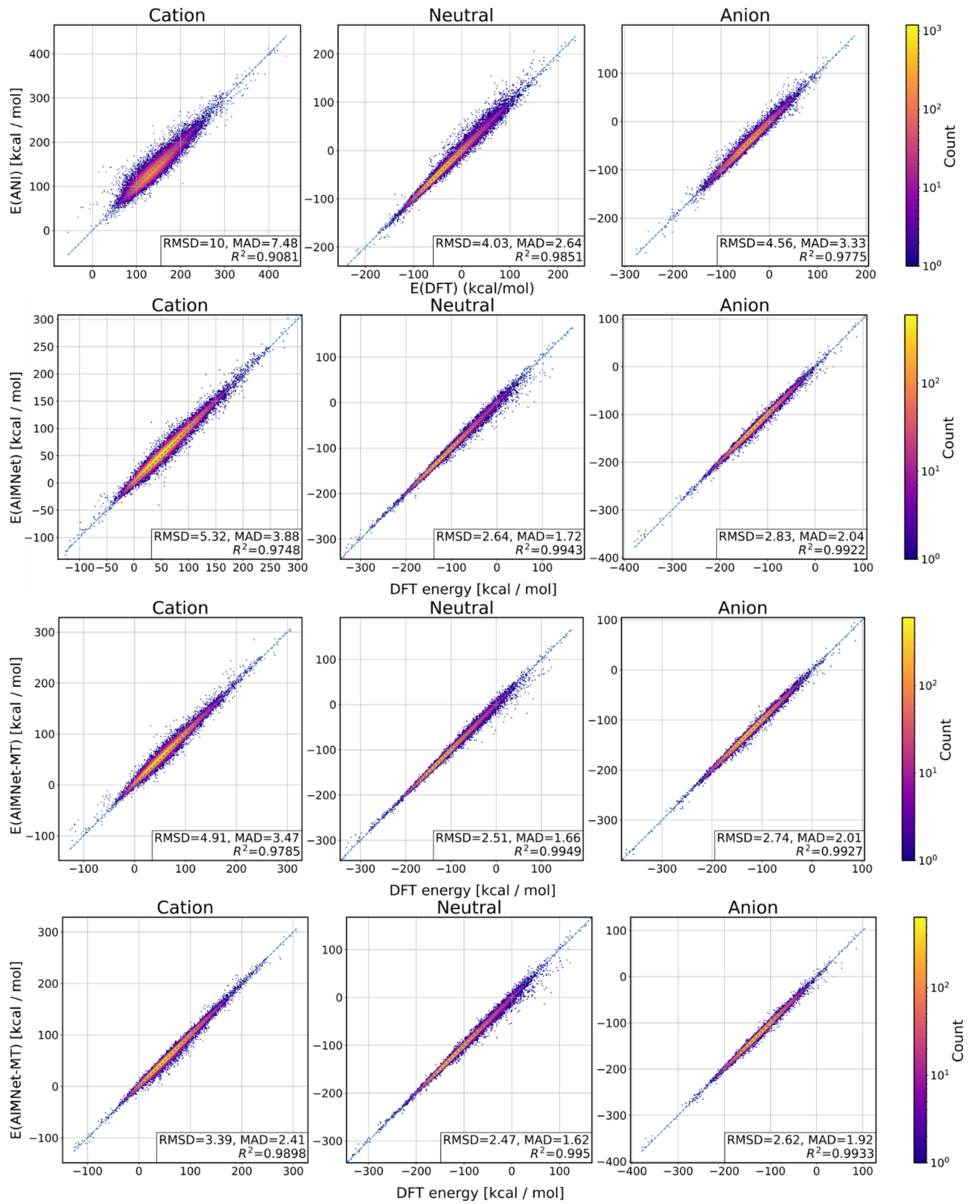

Supplementary Figure 3: Correlation plots for predicted versus DFT total molecular energies for Ions-16 dataset.

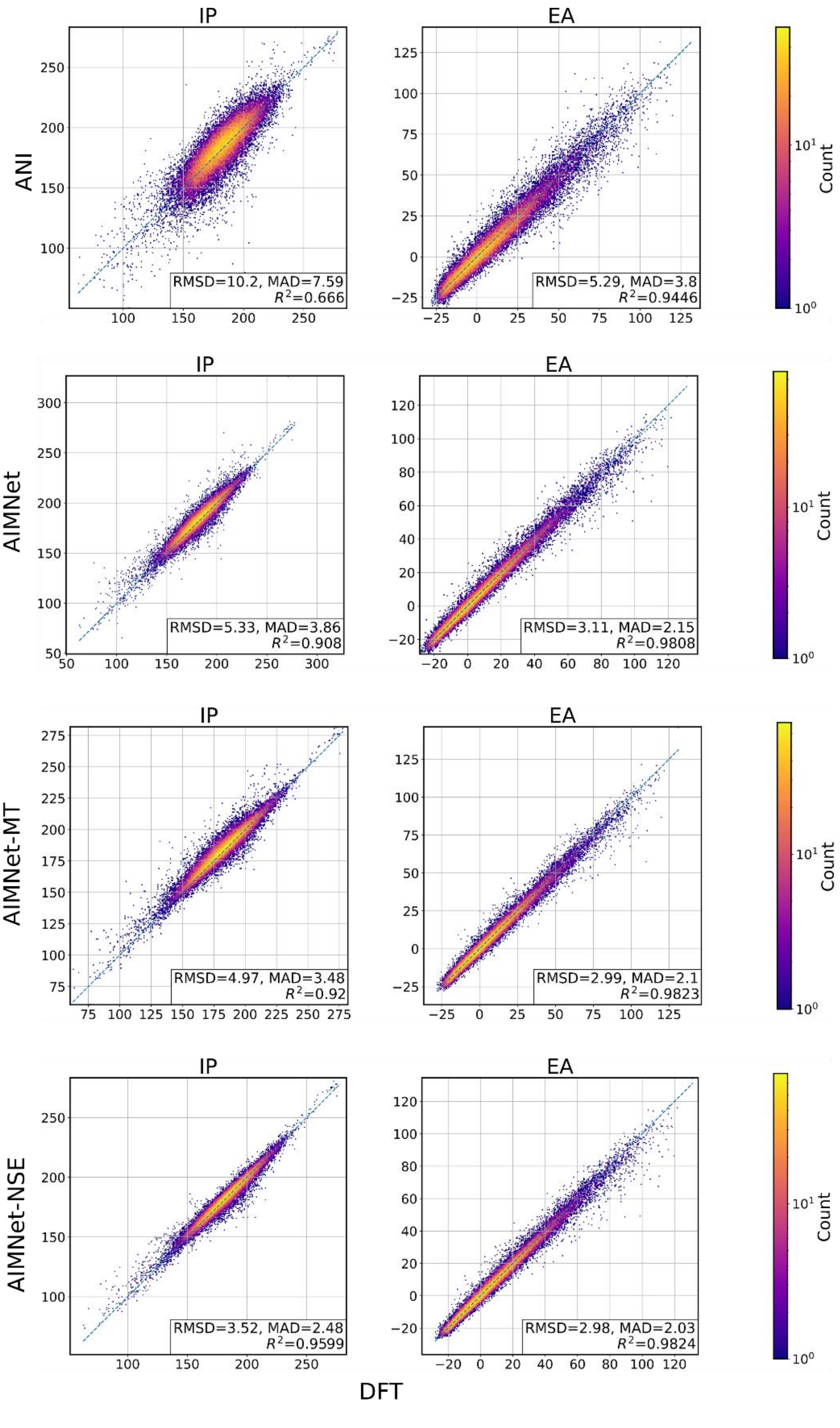

Supplementary Figure 4: Correlation plots for predicted versus DFT ionization potentials (IP) and electron attachment energies (EA) [kcal/mol] for Ions-16 dataset.

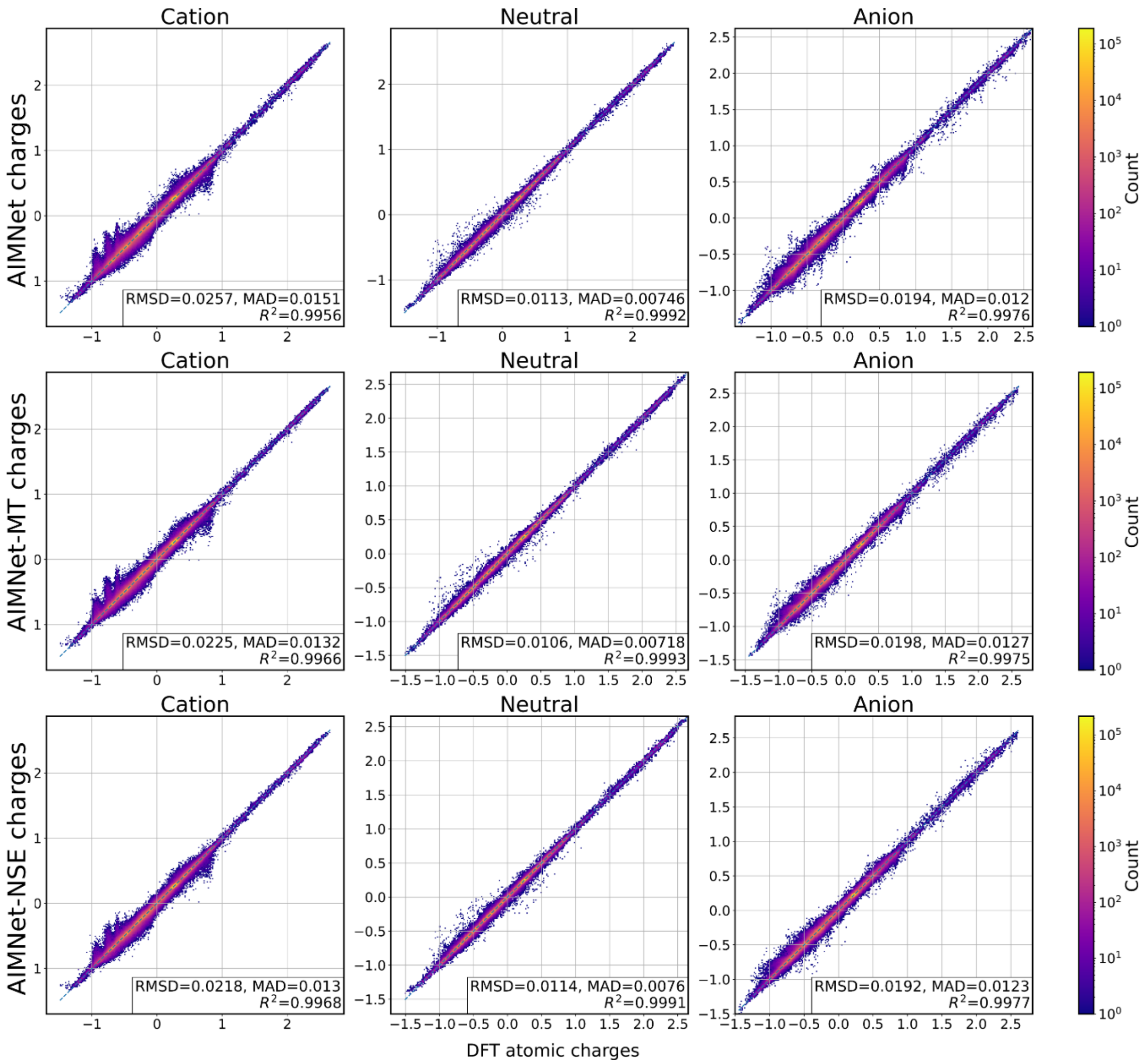

Supplementary Figure 5: Correlation plots for predicted versus DFT NBO partial atomic charges for Ions-16 dataset.
